# Modelling of an Oesophageal Electrode for Cardiac Function Tomography

**DOI:** 10.1155/2012/585786

**Published:** 2012-03-15

**Authors:** J. Nasehi Tehrani, C. Jin, A. L. McEwan

**Affiliations:** CARLAB, School of Electrical and Information Engineering, The University of Sydney, Sydney, NSW 2006, Australia

## Abstract

There is a need in critical care units for continuous cardiopulmonary monitoring techniques. ECG gated electrical impedance tomography is able to localize the impedance variations occurring during the cardiac cycle. This method is a safe, inexpensive and potentially fast technique for cardiac output imaging but the spatial resolution is presently low, particularly for central locations such as the heart. Many parameters including noise deteriorate the reconstruction result. One of the main obstacles in cardiac imaging at the heart location is the high impedance of lungs and muscles on the dorsal and posterior side of body. In this study we are investigating improvements of the measurement and initial conductivity estimation of the internal electrode by modelling an internal electrode inside the esophagus. We consider 16 electrodes connected around a cylindrical mesh. With the random noise level set near 0.05% of the signal we evaluated the Graz consensus reconstruction algorithm for electrical impedance tomography. The modelling and simulation results showed that the quality of the target in reconstructed images was improved by up to 5 times for amplitude response, position error, resolution, shape deformation and ringing effects with perturbations located in cardiac related positions when using an internal electrode.

## 1. Introduction

In electrical impedance tomography (EIT) 16–32 skin contact electrodes are typically connected around the chest and current is injected through pairs of electrodes in a circular shifting format (in the adjacent stimulation pattern) with simultaneous measurement of the voltages on other electrode pairs. Gabriel et al. (1996) calculated and reported that tissues and different organs inside the body display different electrical properties [1]. Due to this fact, EIT is a noninvasive imaging tool with unique information for medical applications. One of the most attractive applications of EIT is functional monitoring of the heart. The contractions of all four chambers of the heart during the cardiac cycle allow selective blood perfusion through lungs and systematic circulation. For the first time McArdle et al. reported that the blood volume circulation changes the conductivity of elements inside the images captured by EIT [2]. There have been several studies on cardiac function EIT where electrodes were arranged around the chest. The study by Brown et al. [3] was one of the first studies using EIT to image cardiac activity. In their study a current (50 kHz and 5 mA) was applied, between all adjacent pairs of 16 electrodes attached on the surface in the transverse plane of the body. This work used a backprojection algorithm which was subsequently found to be inappropriate for EIT as current does not flow in straight lines through the body and online monitoring was not possible with this equipment because of limitations in image reconstruction speed. Edic et al. studied impedance imaging for ventilation and perfusion in human subjects. They used the continuous data acquisition mode of the real-time imaging system (ACT3 system) for collecting data from the thorax of a normal human subject. They reported the admittivity changes in the chest as a result of respiration and the cardiac cycle [4]. Borsic et al. studied simultaneous current injected through 32 current electrodes and measured voltages on 32 voltage electrodes, in a frequency range from 10 kHz to 160 kHz with a realistic thorax model. The image results clearly showed the change of conductivity in the lung regions during respiration. In this method changes of conductivity, which was related to cardiac function, could also be seen in the central region but independent verification of the signals was not possible [5]. Isaacson et al. reported the conductivity changes in cardiac images using nonlinear image reconstruction called D-bar. This method provided reconstructions that could distinguish between different phases of the cardiac cycle [6]. Recently, Solà et al. reported monitoring of central blood pressure. In this study an arterial line was inserted into the ascending aorta for measuring reference BP. EIT images were generated from 32 impedance electrodes placed around the chest at the level of the axilla. The correct location of the aortic ROI was confirmed by hypertonic saline bolus injections directly into the aorta. Aortic pulse transit time (PTT) values were determined as the delay between the opening of the aortic valve and the arrival of pressure pulses at the aortic ROI within the EIT plane [7].

Despite all the efforts on EIT image quality, spatial resolution is still poor. What makes EIT so hard is related to the nonlinear inverse boundary reconstruction, which is highly unstable with respect to the measurement and finite element modeling errors [8, 9]. The effect of the measurement errors mostly relates to the design of the system and electrode connections [10]. Additional common errors of modeling include simplification of the forward model and truncation of real values in the computational domain and unknown boundary data. For example, all of the medical reconstruction methods assume that the boundary of the target body is known. Inaccurate knowledge of the shape of the target body and simplification of the medium in order to decrease the number of elements in the finite element model are other important parameters that contribute to poor images [11].

The method of only arranging electrodes around the medium also could affect the result of the reconstruction especially for cardiac output measurement. Patterson et al. used the Sheffield EIT portable system DAS-01 to determine the change in the cardiac image with electrode position, lung volume, and body position. Results showed significant individual variability with electrode position and air volume. The middle electrode most consistently showed an increase in impedance in the region of the heart during systole. In their study, the pattern of variability with electrode position was not consistent among subjects [12]. The sensitivity of impedance imaging systems to changes in tissue impedance decreases with distance from the nearest electrode (in reality the resolution is best on the periphery and worst at the center) [13]. In a circular array configuration, this means that the central portion of the imaged plane has the least sensitivity. In the cardiac application, the location of the heart between two very high impedance regions composed of lungs, muscles, and bone in the thorax area has a dramatic effect on spatial resolution. It has been proposed that using an internal electrode via the esophagus might improve the quality of the reconstruction. In critical care units, EIT electrodes could potentially be inserted in the esophagus bringing them closer to the heart with a tenfold reduction in their contact impedance in comparison with skin electrodes [1]. One of the early studies on internal electrodes was by Patterson. who estimated stroke volume with thoracic electrical impedance measurements using band electrodes around the neck and lower thorax [14]. Later, Tunstall and Geddes. used esophageal electrodes in dogs to measure respiration. In their report they mentioned observing cardiac generated impedance oscillations [15]. Howeve,r one issue was movement of tissues or the internal electrode. Schuessler and Bates reported a coarse 2D computer simulation, by utilizing an esophageal electrode. They performed two configurations relying entirely on boundary voltages. They reported that the use of esophageal electrode clearly reduced the reconstruction error and noise level [16]. Development of new multifrequency EIT systems such as KHU Mark2 increased the possibility of detecting fast physiological changes during respiration and cardiac activity. The KHU Mark2 is based on an impedance measurement module (IMM) comprising a current source and a voltmeter. This system adopts a pipeline structure that allows the maximum data acquisition speed of 100 frames per second with 16 IMM channels [17]. Recently, we have reported the practical feasibility of using internal electrodes for cardiac EIT by simulating a circulation saline tank similar to normal blood flow inside the heart and in an animal model. In the tank, the movement of the probe was adjustable with a microcontroller and the four terminal impedances measured with a precision impedance analyzer (Agilent 4294A). We found that the internal electrode should be restricted in movements to less than 2 mm. This was achieved in an animal model and the measured transimpedance was correlated with real cardiac impedance changes [18, 19].

In this study we are investigating improvement of the reconstruction and initial conductivity estimation of the internal electrode by modeling this technique in Electrical Impedance Tomography, Diffuse Optical Tomography Reconstruction Software (EIDORS), which is a linear finite element solver written in Matlab. This software was developed to promote collaboration between research groups working on EIT- and diffusion-based optical tomography, in medical and industrial settings [20]. The simulated injection current is 1 mA at 50 kHz, which is an often used and safe current level for EIT at this frequency. This is an imperceptible current for medical applications. The response to these currents is expected to be linear up to several volts on the electrodes where they will then start to become polarised. Images reconstructed by EIT are not well characterized and to compare the methods with each other we use the GREIT (Graz consensus reconstruction algorithm for EIT) [21] and L1 curve evaluation [22].

## 2. Materials and Methods

The EIT problem can be developed from Maxwell's equations. If we consider *Ω* as a given body that is closed and bounded in a subset of 3D space with a boundary of  *δΩ*, *σ* as conductivity, and *z*_*c*_ as contact impedance between the object and the electrodes, then the forward model can be shown as [23]

(1) $$\begin{array}{c} \nabla \cdot \sigma \left(x\right)\nabla u\left(x\right)=0,\;\;x\;\in \;\Omega ,\\ u\left(x\right)+z_{c}\sigma \left(x\right)\frac{\delta u\left(x\right)}{\delta n}=U_{\ell },\;\;x\;\in \;e_{\ell }\in \;\delta \Omega ,\\ \int \sigma \left(x\right)\frac{\delta u\left(x\right)}{\delta n}dS=I_{\ell },\;\;x\;\in \;e_{\ell }\in \delta \Omega ,\\ \sigma \left(x\right)\frac{\delta u\left(x\right)}{\delta n}=0,\;\;\;x\;\in \;\delta \Omega \;∄\;\overset{N_{\text{ el}}}{\underset{\ell =1}{⋃}}e_{\ell }, \end{array}$$

where *u*(*x*) is the voltage distribution in the body and *n* is the outward unit normal vector at  *Ω*. *U*_*ℓ*_ is the voltages measured on the surface of the electrodes and *I*_*ℓ*_ is the current injected through electrodes into the body. *N*_el_ is the number of electrodes connected around the body (in our experiment  *N*_el_ = 16).  *e*_*ℓ*_ is the surface under the electrodes.

The partial differential equations for the forward model converge to a unique solution considering two conditions of conservation of charge ∑_*ℓ*=1_^*N*_el_^ *I*_*ℓ*_ = 0 and choice of a ground  ∑_*ℓ*=1_^*N*_el_^ *U*_*ℓ*_ = 0.

If we simplify the observation model as follows:

(2) $$\begin{array}{c} V=U\left(\delta \sigma \right)+n, \end{array}$$

where *V* is the vector of observation voltage around the boundary, the measurement noise *n* is modeled as Gaussian and independent from conductivity ~*N*(0, *C*_*n*_), and conductivity changes are expressed as  *δσ* ~ *N*(*μ*_*δσ*_, *C*_*δσ*_), then the posterior density can be shown as

(3) $$P\left(\delta \sigma \;|\;V\right)\;\propto \left\{\exp-\frac{1}{2}\times {\left(V-U\left(\delta \sigma \right)\right)}^{T}C_{n}^{-1}\left(V-U\left(\delta \sigma \right)\right)\right\}P\left(\delta \sigma \right).$$

The maximum a posteriori estimate is obtained by maximizing the posterior density, which is identical to the minimization of

(4) $$\begin{array}{cc} \;\varphi_{\text{MAP}}\left(\delta \sigma \right) & =\frac{1}{2}\times {\left(V-U\left(\delta \sigma \right)\right)}^{T}\;C_{n}^{-1}\left(V-U\left(\delta \sigma \right)\right)\\ & \;+\frac{1}{2}\times {\left(\delta \sigma -\delta \sigma^{*}\right)}^{T}C_{\delta \sigma }^{-1}\left(\delta \sigma -\delta \sigma^{*}\right), \end{array}$$

where δσ* is a prior estimate of the distribution of conductivity changes.

Equation (4) leads to the following minimization problem mostly recognized as L2 norm or the Tikhonov regularization method [24–26]:

(5) $$\begin{array}{c} \text{argmin}\left\{{||L_{n}\left(V-U\left(\delta \sigma \right)\right)||}^{2}+{||L_{\delta \sigma }\left(\delta \sigma -\delta \sigma^{*}\right)||}^{2}\right\}, \end{array}$$

where *C*_*n*_^−1^ = *L*_*n*_^*T*^ *L*_*n*_, and *C*_*δσ*_^−1^ = *L*_*δσ*_^*T*^ *L*_*δσ*_.

### 2.1. Finite Element Model and Simulation

**Figure 1:**
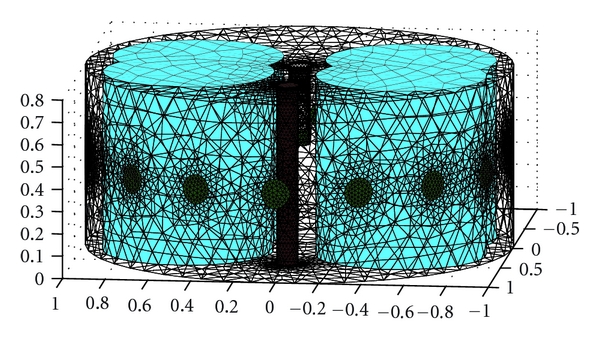
Cylindrical model of the thorax with electrode number 16 located in the middle of the cylinder as an esophageal electrode and surrounded by compound cylindrical models of the left and right lungs. The other 15 electrodes are connected on the external surface of the cylinder. The perturbation with the conductivity of the blood is located 0.5 units from the center of the cylinder.

**Figure 2:**
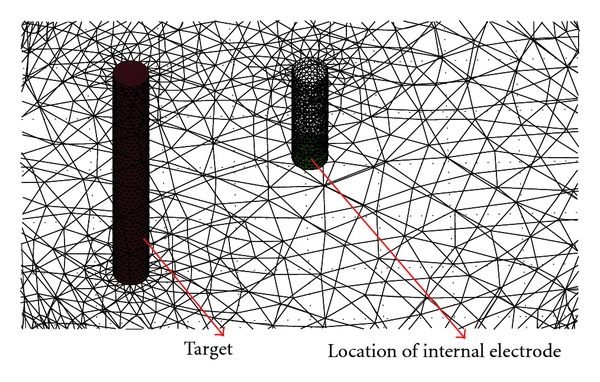
This figure shows large size of mesh for internal cylinder used for internal electrode and its relation with the perturbation.

The common method for solving the partial differential equation in EIT is the finite element method (FEM) [27, 28]. We used 3D forward and inverse models as 2D models could not account for the true distribution of current in the medium. For 3D FEM the domain is first divided into small tetrahedral elements. The density of elements in the FEM model will define the accuracy of the simulation. For evaluating the reconstructed images with parameters explained in GREIT, it is recommended that the model should have a number of elements more than 25000 [21]. The FEM model used in this study was cylindrical and surrounded by circular electrodes (Figure 1). The dimensions were related to our saline tank setup with the following relative scales: the boundary cylinder had a diameter of 2 units and height 0.8 units (*D* = 2, *Z* = 0.8), with circular electrodes (*D* = 0.1). An empty cylinder in the middle of the boundary cylinder was the location of the internal electrode with the same diameter as normal electrodes and height of the entire boundary (*D* = 0.1, *Z* = 0.8). We used the same size of the electrodes for internal and boundary electrodes to decrease variability in contact impedance between electrodes. All electrodes were located with their centers at  *Z* = 0.4. The red perturbation inside the medium (Figure 1) with the conductivity of the heart blood had a cylindrical shape (*D* = 0.1,  *Z* = 0.8) and the conductivity of left and right lungs was modeled as a two-compartment cylindrical shape in blue (*D* = 0.8, *Z* = 0.8). We adopted widely used values for the conductivity of the blood and the inflated lungs at a frequency of 50 kHz (http://niremf.ifac.cnr.it/tissprop/) [29]. The conductivity of the blood in our model is 0.7 s m^−1^, the lung inflated is 0.13 s m^−1^ while other chest tissue is assigned a conductivity of 0.48 s m^−1^.  Figure 2 shows a typical size and location of the internal perturbation relative to the internal electrode. We moved the red cylinder as a small variant related to the blood volume changes during cardiac cycles from the center of the cylinder very close to the esophagus to the front side of the cylinder (apex of the heart) to compare the results of new arrangement of electrode (internal electrode) with external arrangement of electrodes. Since increasing the mesh resolution decreases the error in the calculated data, we implemented the same resolution for both methods [30]. For the forward model we used a fine mesh with 37409 elements and 8162 nodes and for the inverse problem we used coarser mesh with 28041 elements and 3210 nodes. We calculated the simulated voltages using a forward model with a different mesh than the one used for image reconstruction to avoid the so-called “inverse crime” [31].

For the normal simulation all 16 electrodes were located around the cylinder and for the model with internal electrode we moved the electrode number 16 inside the cylinder. The ground electrode for both simulations was considered on the bottom and middle of the cylinder. Simulation of thorax as a cylindrical model is reasonable for EIT simulation and easily can be experimented with phantom tanks [32]. The FEM we used in this study was generated using NETGEN software [33]. The reconstructed images were exported from Matlab to MayaVi (http://mayavi.sourceforge.net/) for data visualization.

### 2.2. Evaluated Parameters

EIT methods for image reconstruction can be evaluated by the following parameters that describe the quality of the reconstructed images. Amplitude response (AR) measures the ratio of image pixel amplitudes in the target to that in the reconstructed image. Position error (PE) measures the distance error between the center of the gravity (COG) in the target and the reconstructed image. Resolution (RES) measures the size of reconstructed targets (number of pixels) as a fraction of the whole reconstructed pixel area. In reconstructed images of EIT the target is often surrounded with impedance changes in the opposite direction of the simulated target, which is called overshoot or ringing. This can also be interpreted as the point spread function (PSF). Shape deformation (SD) measures the artefacts of the reconstructed image with the real target.

**Figure 3:**
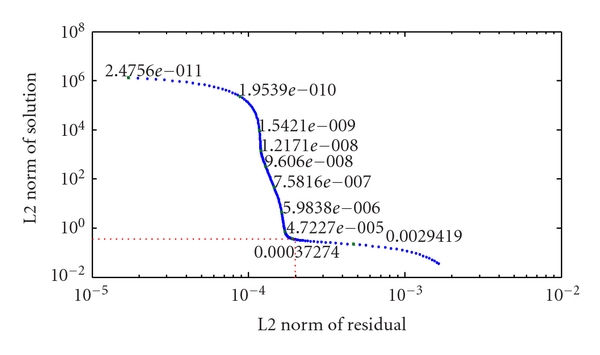
A typical curve for the inverse problem showing iterative convergence to the sparse solution. The numbers shown on the L-curve are *λ* (regularization parameter) [32].

Often the regularization parameter is adjusted manually. Here for a fair comparison between the images of the methods we used automatic selection of the optimum regularization parameter as the L-corner of the L-curve.  Figure 3 shows a typical solution for the inverse problem using the Tikhonov regularization algorithm. This curve visualizes the trade-off between the norms of the residual  (*V* − *U*(*δσ*)) and the solution (*δσ*) with different regularization parameters. The red dashed line on the curve shows the L-corner, which is the best trade-off between residual and solution in inverse problems (optimum regularization parameter). The good regularization parameter *λ* is one that corresponds to a regularized solution near the corner of the L-curve because in this region there is a good compromise between achieving a small residual norm and keeping the solution seminorm reasonably small [34]. We report the regularization parameter at the L-corner as the regularization parameter seeks to trade off resolution and conditioning of the inverse solution [35]. We expect the internal electrodes to provide improved information over the normal, boundary electrode arrangement and so expect that the regularisation parameter is smaller in this case demonstrating a more robust imaging approach [36]. For more details on the parametes see [21, 22]. To examine cardiac related positions, the perturbation was moved from 0.2 units from center of the cylinder in 0.1 unit increments to 0.8 units from the center towards electrode number 9.

For evaluating the internal electrode with the GREIT parameters we evaluate the situation where we have small changes related to blood conductivity (e.g., point spread function). This does not affect the main concept as our region of interest is the cardiac location, and we assume the ventilation can be controlled in the critical care unit to prevent lung changes. For a fair comparison between methods this removes the nonlinearity related to the shape of the lungs and also rules out any possible effects on the evaluated parameters caused by lung artefacts. In our evaluation the target was located 0.2 units from center of the cylinder and each time the distance from the center increased by 0.1 unit in the direction of electrode 9.

## 3. Results and Discussion

### 3.1. Finite Element Model and Simulation

**Figure 4:**
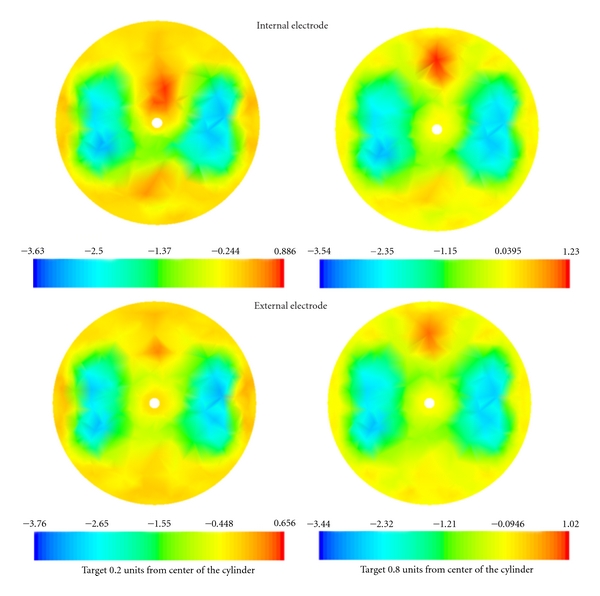
The reconstruction of methods for the blood perturbation located 0.2 units (left column) and 0.8 units (right column) from the center. For the reconstruction on the top row electrode number 16 was used as an internal electrode and for the reconstructions on the bottom row all electrodes were located around the cylinder. The perturbation with blood conductivity is shown in red and the left and right lung conductivities are shown in blue.

Figure 4 shows the result of image reconstruction for the blood perturbation with diameter of  0.2  (*r* = 0.1). The left column shows the result when the blood perturbation is located 0.2 units from the center of the cylinder (close to the oesophagus) and the right column shows the result when the blood perturbation was located 0.8 units from the center of the cylinder (the apex of the heart near). Images in the top row used an internal electrode and images in the bottom row used an external arrangement of the electrodes. On the bottom of each reconstruction a colour scale represents the intensity of the reconstructed signal inside of the cylinder. The blood changes located 0.2 units from the center are hardly detected with external arrangement of electrodes (bottom row) when surrounded with high resistivity area of left and right lungs. Figure 4 also shows that the intensity of the reconstruction improves by factor 1.2 for the changes 0.8 units from the center by using the internal electrode (top row).

### 3.2. Evaluated Parameters

**Figure 5:**
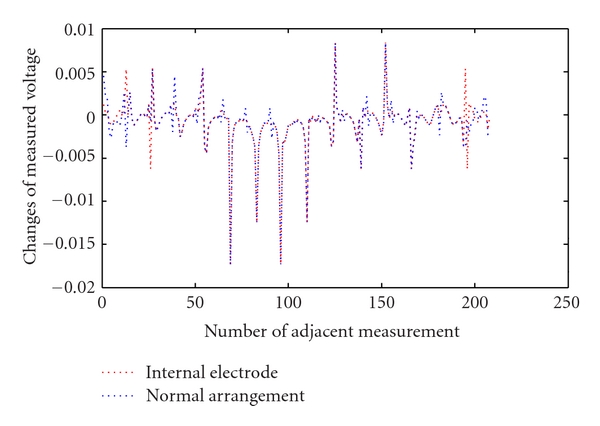
208 measured voltage around two different models changes of voltages related to 0.05 units increase in radius of perturbation with  *r* = 0.3.

With 16 electrodes and an adjacent stimulation pattern the number of measured voltages for each frame is  16 × 13 = 208. The typical simulated voltages related to one frame have been shown in Figure 5. This graph is the result of an increment of 0.05 units in the radius of the cylindrical perturbation with radius of 0.3 units (close to the heart size changes during ECG gating) located 0.5 units from the center of the medium. The U-shaped pattern of the graph shows that as we move away from the current electrodes (starting at adjacent measurement 1) the measured voltages will decrease. The voltage increases again as we move back to the current electrodes then the pattern repeats 16 times. In Figure 5, it is clear that in some measurement combinations the voltages measured with the internal electrode are amplified particularly around the voltages that relate to the internal electrode 16 (near 200 and less than 50).

**Figure 6:**
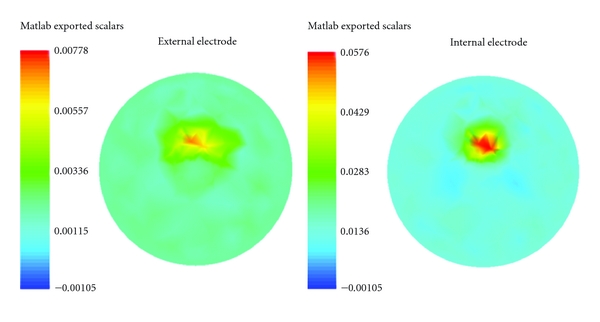
Left image shows the reconstruction of target with external electrode arrangement, and right image shows the reconstruction with internal electrode. The target located 0.2 units from center of the cylinder.

Figure 6 shows typical reconstruction image of perturbation located 0.2 units from the center of the cylinder. On the left side of reconstructions a colour scale represents the intensity of the reconstructed signal inside of the cylinder. Qualitatively, all reconstructed images with internal electrodes show that the ringing effect around the target decreases and the solution seminorm is smaller than external electrodes. Both internal electrode and normal arrangement images show that the reconstruction improves for the images where the target is closer to the electrodes.

**Figure 7:**
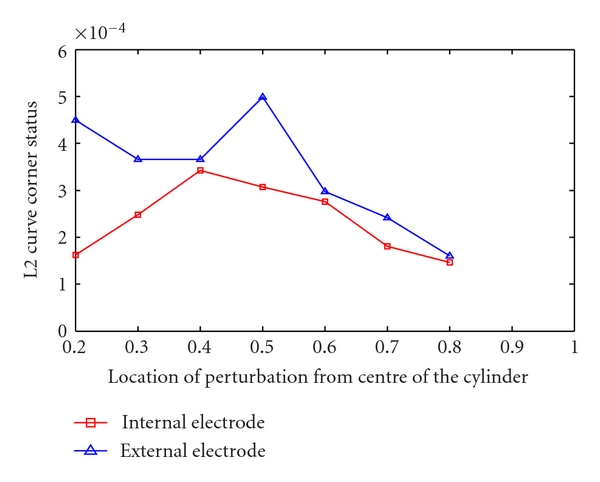
The red curve shows the L-corner (optimum regularization parameter) of the solution with internal electrode and the blue curve shows the L-corner of the solution with normal arrangement.

The change in the L-curve and thus the robustness of the solutions with position changes is demonstrated in Figure 7. This curve shows that the optimum regularization parameter (L-corner) of the solution for the internal electrode is always less than the normal arrangement, and particularly lower for central locations. For both arrangements of the electrodes the robustness decreases for the targets located midway from the center of the cylinder, which are the furthest locations from the electrodes. The optimum regularization parameter (L-corner) for the internal electrode increases then returns to a similar value for locations near the center and boundary implying that the reconstruction results in central regions have a robustness similar to perturbations on the boundary.

**Figure 8:**
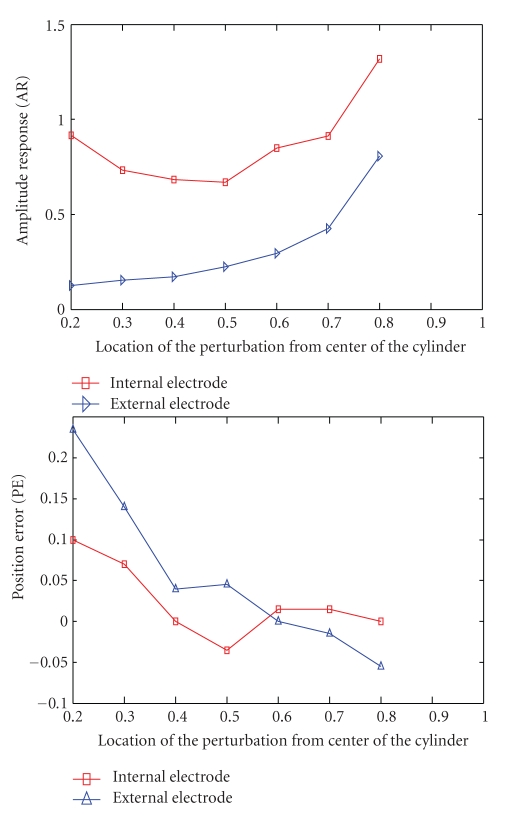
The right curve shows the amplitude response and the left one shows the position error of two methods. The red curve relates to the internal electrode modeling and the blue curve is the normal arrangement.

In Figure 8 the amplitude response of the method with internal electrode shows a better intensity in comparison with the normal arrangement at all locations. In general, the best AR is achieved when the targets are close to the electrodes. For that reason the AR for the internal electrode has a minimum AR near the midway point when the target is furthest from the electrodes and the optimum locations are central and boundary. For the external electrodes arrangement the AR is increased by moving the target from the center of the cylinder (i.e., the furthest point from all electrodes) to the boundary.

For position error also the curves show that the maximum PE for internal electrode simulation is in the middle near 0.5 and the PE improves near the electrodes. For the external electrodes arrangement the maximum PE is for the target near the middle of the cylinder, which is the furthest location from all electrodes. The PE curves for both methods show a convergence when the target moves to the boundary electrodes with a small improvement for the method with internal electrode relative to the external electrodes case.

**Figure 9:**
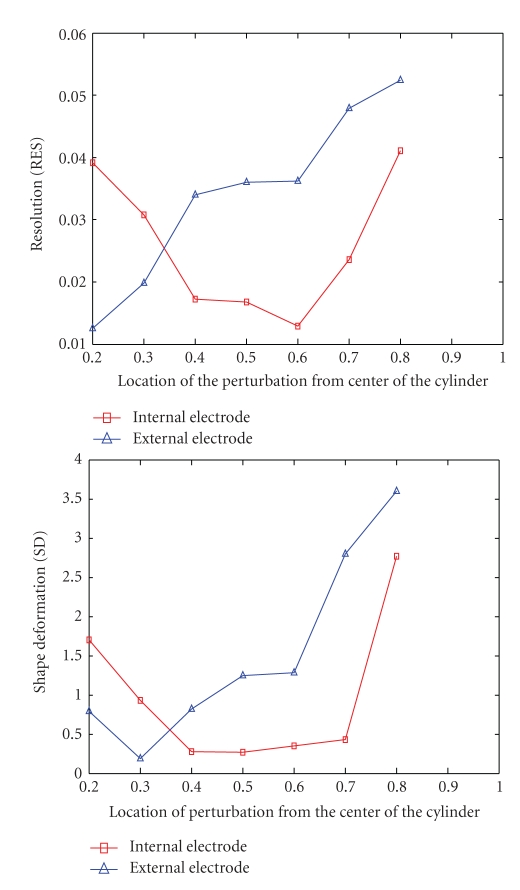
The right curve shows the resolution and the left one shows the shape deformation.

In Figure 9 the curves related to the resolution and shape deformation have been shown. As with the other evaluated parameters, resolution improved when the object moved close to the electrodes; however, shape deformation also increased near the internal electrode. The flat curve around 0.4 to 0.7 units from the center is because the number of mesh elements selected has increased leading to smoothing of the target in the reconstructed image [30]. This is possibly due to incorrect interpretation of images or effects of electrode artefacts, which are sometimes caused by a higher sensitivity near the electrodes. The optimum point for shape deformation with the internal electrode was midway at around 0.5 units from the center where the perturbation is furthest from any electrode. We expect to see cardiac related changes in this area where the blood is exchanged with the lungs, so in Table 1 we show a comparison chart of this situation with target size of *D* = 0.1 unit.

### 3.3. Clinical Impact

The current gold standard in critical care units for cardiac function measurement is a gated heart pool scan, which provides a good assessment of global heart function, but with low resolution, long lead time, and radiation risks. Less accurate alternatives include transoesophageal echocardiography, where the ultrasonic probe is inserted into the oesophagus behind and close to the heart, and transthoracic echocardiography, where the probe observes the heart through the intercostal spaces. These echo modalities often do not provide satisfactory results in these patients due to lung and rib artefact, and the size of probe currently used for the transoesophagus echocardiogram is large, inconvenient for patients, and unable to be left indwelling.

In critical care units mostly the subject is ventilated and the lungs function could pause for impedance measurements. So we expect that the artefacts of lungs movement decrease significantly in this area.

Our study shows that using an internal electrode for cardiac function imaging can improve the GREIT evaluation parameters such as AR, PE, RES, and SD in reconstructed images. The internal electrode can provide more information about the inner area of the heart in comparison with the external arrangement of electrodes. EIT is a fast method for global cardiac output imaging in this area. A stainless steel internal electrode similar to the ablation catheters is smaller than ultrasonic probes and can easily be inserted through oesophagus and remain indwelling for significant periods of time.

Two practical issues that need further consideration in developing this method are safety and artefacts generated by the catheter movement.

Regarding safety, several groups, including our own, have performed practical impedance measurements with oesophagus electrodes in humans and animals [14–16, 18]. To the best of our knowledge there have been no reports of ill effects from using internal electrodes as voltage sensing or current injection electrodes with currents up to 5 mA at 50 kHz and in our measurements we did not find any influence on additional monitoring devices (such as ECG monitoring), that would normally be expected in the critical care unit [18]. However, we recommend that additional analysis is required here to examine the safety aspects as further increases in current is desirable as they will lead to improved signal to noise ratio and improved images.

Regarding movement artefacts, we have modelled the location of the internal electrode in the plane of external ones and suggest that this can be confirmed in practice by other imaging methods such as fluoroscopy. As we insert the internal electrode close to heart, the internal electrode movements related to the heart are a significant artefact that should be carefully considered. In previous practical experiments we tried the oesophagus and superior vena cava (SVC) electrodes. Our evaluation shows that the electrode located in SVC is less affected by the heart movement and this electrode could be used as a reference to remove the artifacts generated by movements in the esophagus internal electrode.

## 4. Conclusion

There is a need in critical care units for continuous cardiopulmonary monitoring techniques. One attractive technique is EIT. In critical care units specialists have access to the esophageal area through catheters and previously practical measurements indicated that the contact impedance of electrodes is decreased in this area [1]. In addition, by inserting an extra electrode in this area right behind the heart we aim to reduce the high impedance areas related to the lungs and muscles. ECG-gated EIT has the potential to localize the impedance variations occurring during the cardiac cycle. In this study we investigated the improvement of the EIT image reconstruction by implementing an internal electrode, and we compare the results with the current electrode arrangement. This study was performed in Matlab by simulation of the thorax as a cylindrical model. Partial differential equations were solved for forward and inverse problems by using EIDORS. For evaluation of the new arrangement of electrodes we used GREIT agreed parameters for EIT reconstructed image comparison, which have previously been used for reconstruction of lung images. Our evaluation showed that using the internal electrode improved the resolution, amplitude response, seminorm of solution, and position of the target in the center of the image to that normally measured on the periphery. Shape deformation showed an improvement in the region at 0.5 units from the center. Overall this evaluation demonstrated an improvement in the results with internal electrodes particularly in cardiac related regions located centrally in the body of interest. For these reasons, we recommend further exploration and use of internal electrodes in cardiac EIT.

## Figures and Tables

**Table 1:** Comparison of initial distribution based on electrode arrangement. The target located at 0.5 units.

	AR	PE	RES	SD	L corner
Normal arrangement	0.23	0.045	0.036	2.59	0.00049
Internal electrode	0.61	0.035	0.0168	0.68	0.00030

## References

[1] Gabriel S, Lau RW, Gabriel C (1996). The dielectric properties of biological tissues: II. Measurements in the frequency range 10 Hz to 20 GHz. *Physics in Medicine and Biology*.

[2] McArdle FJ, Suggett AJ, Brown BH, Barber DC (1988). An assessment of dynamic images by applied potential tomography for monitoring pulmonary perfusion. *Clinical Physics and Physiological Measurement A*.

[3] Brown BH, Leathard A, Sinton A, McArdle FJ, Smith RW, Barber DC (1992). Blood flow imaging using electrical impedance tomography. *Clinical Physics and Physiological Measurement A*.

[4] Edic PM, Saulnier GJ, Newell JC, Isaacson D (1995). A real-time electrical impedance tomograph. *IEEE Transactions on Biomedical Engineering*.

[5] Borsic A, McLeod C, Lionheart WRB, Kerrouche N (2001). Realistic 2D human thorax modelling for EIT. *Physiological Measurement*.

[6] Isaacson D, Mueller JL, Newell JC, Siltanen S (2006). Imaging cardiac activity by the D-bar method for electrical impedance tomography. *Physiological Measurement*.

[7] Solà J, Adler A, Santos A, Tusman G, Sipmann FS, Bohm SH (2011). Non-invasive monitoring of central blood pressure by electrical impedance tomography: first experimental evidence. *Medical and Biological Engineering and Computing*.

[8] Kaipio J, Somersalo E (2007). Statistical inverse problems: discretization, model reduction and inverse crimes. *Journal of Computational and Applied Mathematics*.

[9] Kolehmainen V, Lassas M, Ola P (2005). The inverse conductivity problem with an imperfectly known boundary. *SIAM Journal on Applied Mathematics*.

[10] McEwan A, Cusick G, Holder DS (2007). A review of errors in multi-frequency EIT instrumentation. *Physiological Measurement*.

[11] Nissinen A, Kolehmainen VP, Kaipio JP (2011). Compensation of modelling errors due to unknown domain boundary in electrical impedance tomography. *IEEE Transactions on Medical Imaging*.

[12] Patterson RP, Zhang J, Mason LI, Jerosch-Herold M (2001). Variability in the cardiac EIT image as a function of electrode position, lung volume and body position. *Physiological Measurement*.

[13] Kerner TE, Williams DB, Osterman KS, Reiss FR, Hartov A, Paulsen KD (2000). Electrical impedance imaging at multiple frequencies in phantoms. *Physiological Measurement*.

[14] Patterson RP (1987). Possible technique to measure ventricular volume using electrical impedance measurements with an oesophageal electrode. *Medical &amp; Biological Engineering &amp; Computing*.

[15] Tunstall ME, Geddes C (1984). “Failed intubation” in obstetric anaesthesia. An indication for use of the “esophageal gastric tube airway”. *British Journal of Anaesthesia*.

[16] Schuessler TF, Bates JHT Utility of an esophageal reference electrode for thoracic electrical impedance tomography.

[17] Oh TI, Wi H, Kim DY, Yoo PJ, Woo EJ (2011). A fully parallel multi-frequency EIT system with flexible electrode configuration: KHU Mark2. *Physiological Measurement*.

[18] Nasehi-Tehrani J, Chik W, Barry MA 3D EIT for cardiac function imaging using internal electrodes: preliminary simulation and pilot results.

[19] Nasehi-Tehrani J, Thiagalingam A, Jin C Feasibility of using internal electrodes to improve the accuracy cardiac electrical impedance tomography.

[20] Adler A, Lionheart WRB (2006). Uses and abuses of EIDORS: an extensible software base for EIT. *Physiological Measurement*.

[21] Adler A, Arnold JH, Bayford R (2009). GREIT: a unified approach to 2D linear EIT reconstruction of lung images. *Physiological Measurement*.

[22] Nasehi-Tehrani J, McEwan A, Jin C, van Schaik A (2012). L1 regularization method in electrical impedance tomography by using the L1-curve (Pareto frontier curve). *Applied Mathematical Modelling*.

[23] Cheney M, Isaacson D, Newell JC (1999). Electrical impedance tomography. *SIAM Review*.

[24] Vauhkonen M, Vadâsz D, Karjalainen PA, Somersalo E, Kaipio JP (1998). Tikhonov regularization and prior information in electrical impedance tomography. *IEEE Transactions on Medical Imaging*.

[25] Tikhonov A (1963). Solution of incorrectly formulated problems and the regularization method. *English Translation of Dokl Akad Nauk SSSR*.

[26] Kim KY, Kim BS, Kim MC, Kim S (2004). Dynamic inverse obstacle problems with electrical impedance tomography. *Mathematics and Computers in Simulation*.

[27] Sahalos JN The electrical impedance of the humanhead by using a 3-D FEM model.

[28] Woo EJ, Hua P, Webster JG, Tompkins WJ (1994). Finite-element method in electrical impedance tomography. *Medical and Biological Engineering and Computing*.

[29] Gabriel C, Gabriel S, Corthout E (1996). The dielectric properties of biological tissues: I. Literature survey. *Physics in Medicine and Biology*.

[30] Dehghani H, Soleimani M (2007). Numerical modelling errors in electrical impedance tomography. *Physiological Measurement*.

[31] Lionheart WRB (2004). EIT reconstruction algorithms: pitfalls, challenges and recent developments. *Physiological Measurement*.

[32] Patterson RP, Zhang J (2003). Evaluation of an EIT reconstruction algorithm using finite difference human thorax models as phantoms. *Physiological Measurement*.

[33] Schoberl J (1997). NETGEN: an advancing front 2D/3D mesh generator based on abstract rules. *Computing and Visualization in Science*.

[34] Hansen PC (1992). Analysis of discrete Ill-posed problems by means of the L-curve. *SIAM Review*.

[35] Adler A, Guardo R (1996). Electrical impedance tomography: regularized imaging and contrast detection. *IEEE Transactions on Medical Imaging*.

[36] Woo EJ, Hua P, Webster JG, Tompkins WJ (1993). Robust image reconstruction algorithm and its parallel implementation in electrical impedance tomography. *IEEE Transactions on Medical Imaging*.

